# A systematic review of evidence for fitness-to-drive among people with the mental health conditions of schizophrenia, stress/anxiety disorder, depression, personality disorder and obsessive compulsive disorder

**DOI:** 10.1186/s12888-017-1481-1

**Published:** 2017-08-31

**Authors:** Carolyn A. Unsworth, Anne M. Baker, Man H. So, Priscilla Harries, Desmond O’Neill

**Affiliations:** 1 Central Queensland University, Melbourne, Australia; 2 Australian Catholic University, Melbourne, Australia; 3 Brunel University London, London, UK; 4 Trinity College, Dublin, Republic of Ireland

**Keywords:** Fitness-to-drive, Mental health, Systematic review

## Abstract

**Background:**

Limited evidence exists regarding fitness-to-drive for people with the mental health conditions of schizophrenia, stress/anxiety disorder, depression, personality disorder and obsessive compulsive disorder (herein simply referred to as ‘mental health conditions’). The aim of this paper was to systematically search and classify all published studies regarding driving for this population, and then critically appraise papers addressing assessment of fitness-to-drive where the focus was not on the impact of medication on driving.

**Methods:**

A systematic search of three databases (CINAHL, PSYCHINFO, EMBASE) was completed from inception to May 2016 to identify all articles on driving and mental health conditions. Papers meeting the eligibility criteria of including data relating to assessment of fitness-to-drive were critically appraised using the American Academy of Neurology and Centre for Evidence-Based Medicine protocols.

**Results:**

A total of 58 articles met the inclusion criteria of driving among people with mental health conditions studied, and of these, 16 contained data and an explicit focus on assessment of fitness-to-drive. Assessment of fitness-to-drive was reported in three ways: 1) factors impacting on the ability to drive safely among people with mental health conditions, 2) capability and perception of health professionals assessing fitness-to-drive of people with mental health conditions, and 3) crash rates. The level of evidence of the published studies was low due to the absence of controls, and the inability to pool data from different diagnostic groups. Evidence supporting fitness-to-drive is conflicting.

**Conclusions:**

There is a relatively small literature in the area of driving with mental health conditions, and the overall quality of studies examining fitness-to-drive is low. Large-scale longitudinal studies with age-matched controls are urgently needed in order to determine the effects of different conditions on fitness-to-drive.

**Electronic supplementary material:**

The online version of this article (10.1186/s12888-017-1481-1) contains supplementary material, which is available to authorized users.

## Background

The ability to drive enables access to the community, services, friends and family, and therefore promotes autonomy and social connectivity [1, 2]. Driving is a complex task that demands a wide range of skills, abilities and behaviours [3]. Driving involves physical, cognitive, and perceptual skills, along with the ability to respond to the external environment. Driving can be influenced by extent of past experience and personality characteristics [4]. There is considerable debate about the driving ability of people with mental health conditions. The fitness-to-drive of people with mental health conditions may vary within individuals due to both the effects of the illness itself, as well as the impact of psychiatric drugs on driving performance. Individuals diagnosed with mental health conditions may experience reduced attention, visual spatial functioning, impulse control, judgement, as well as alterations in information processing ability and slowed psychomotor reaction times [5–7]. These difficulties can all impair driving abilities [8, 9] and may lead to a recommendation not to drive, a restricted driving licence, driving suspension or licence cancellation [10]. It is important that any restriction is based on evidence to the greatest extent possible, and reflects a due balance between mobility and safety [11] as driving cessation is associated with increased levels of depression [12].

The proportion of individuals with mental health conditions who drive is unknown. However, it is believed that a significant proportion of people are driving, and able to do so safely. For example, 44% of people with mental health conditions from regional and 35% from metropolitan areas in Australia were reported as active drivers [13]. Some studies [14–16] suggest that drivers with mental health conditions have a higher risk of being involved in a crash. However, assessment to determine fitness-to-drive of individuals with mental health conditions is difficult because of the fluctuating nature of impairments, skills and behaviours [17–19]. Currently, there is no single assessment that can be used to accurately predict driving ability of people with mental health conditions [20]. Instead, medical and occupational therapy assessments, neuropsychological tests, and performance based assessments such as on-road assessment and car driving simulator tests are commonly used to determine if a person is fit-to-drive. However, medical guidelines have been developed internationally, and are regularly updated, to assist health professionals in determining fitness-to-drive. These medical guidelines can be used to provide recommendations on licence renewal, suspension or cancellation for clients with mental health conditions. Existing guidelines include those produced by the Driver and Vehicle Licensing Agency (DVLA) in the UK [21], the New Zealand Transport Agency [22], the Irish Road Safety Authority [23], Austroads in Australia [10], Driver Fitness Medical Guidelines in the USA [24] and the Canadian Council of Motor Transport Administrators [25]. While all these provide general information on health conditions as well as guidance and recommendations on assessing fitness-to-drive, only the DVLA [21] guidelines provide clear and detailed recommendations on minimum stand-down periods from driving relating to various psychiatric conditions although the evidence base for these recommendations is unknown, as is the extent to which clinicians comply with them.

The driving literature is dominated by research investigating the impact of physical and cognitive problems arising from dementia and acquired disorders such as brain injury (including stroke), on driving. While there is a growing literature on the impact of attention deficit hyperactivity disorder on driving, evidenced by several systematic review [26–30], only two literature reviews summarising evidence of fitness-to-drive and the mental health conditions of schizophrenia, stress/anxiety disorder, depression, personality disorder and obsessive compulsive disorder have been published [31, 32]. Tsuang, Boor, and Fleming (1985) [31] reviewed the literature in order to identify risk factors related to psychopathology and personality variables for crashes. The authors suggested that the studies reviewed were insufficiently homogenous to understand these relationships. A subsequent systematic review conducted by Menard and Korner-Bitensky (2008) [32] sought to identify and appraise published studies on fitness-to-drive amongst people with mental health conditions and the effect of psychotropic medications on driving performance. This review included 14 studies, all published before 1990. The authors concluded that there was no consistent evidence to support the hypothesis of an increased crash rate amongst drivers with severe mental health conditions. For those with milder or better controlled mental health conditions, conclusions were more difficult to draw given contradictory evidence about fitness-to-drive. Since it was assumed that many new studies would have been published on the topic of mental health and driving since 1990, it was timely to review this literature and appraise current evidence regarding the fitness-to-drive with this population.

We specifically aimed to critically appraise literature on driving among people with the mental health conditions of schizophrenia, stress/anxiety disorder, depression, personality disorder and obsessive compulsive disorder, as anecdotally clinicians most frequently ask us questions about these groups. However, we also made the decision to exclude driving literature with these populations when the focus was specifically on the impact of medication on driving given the estimated size of this literature would require a separate review and our primary interest was in how the medical conditions impact on driving, regardless of pharmacological interventions. We were particularly influenced by the fact that many clients are stable on medication when driving is reviewed, and also the growing literature which suggests that skills for driving may be impaired in people with mental health conditions, and this decrement is not related to the effects of medications [6, 17]. In addition, we did not include literature that examined the driving of people with attention deficit hyperactivity disorder (ADHD). Although many of the mental health conditions included in this review such as anxiety, depression, or personality disorder, are commonly comorbid to ADHD, we chose to exclude this literature based again on its size and that five systematic reviews have already summarised this literature [26–30]. However, it is important to note that many of the problems experienced by people with mental health conditions that may impair driving, such as impulsivity, reduced response inhibition, reduced situational awareness, and the fluctuating nature of the condition, are also experienced by people with ADHD [27]. Therefore, the Discussion section does make reference to this literature.

### Aim

The broad aim of this systematic review was to identify what is known about driving for people with mental health conditions, and critically appraise studies that empirically investigated assessment of fitness-to-drive among people with mental health conditions. Specifically the review aimed to answer the questions:

1. What is the scope of published literature on the topic of mental health and driving (excluding drivers with ADHD), and
2. What is the scope and quality of empirical studies addressing assessment of fitness-to drive among people with mental health conditions, where the focus is not on the impact of medication on driving.

## Methods

This systematic review followed PRISMA guidelines [33], and the protocol for this review is available from the corresponding author.

### Search strategy

Three electronic databases (CINAHL, PSYCHINFO, EMBASE) were used to identify articles from inception to 10th May, 2016. Extensive search terms were identified and used to reduce the risk of missing relevant studies. Search terms included: automobile driv*, driv* ability, driv* competence, driv* perform*, driv* skill, fit*to driv*, AND affective, alcohol abus*, alcohol dependence, alcohol misuse*, alcohol use*, anxiet*, anxious, bipolar disorder, delusion, depress*, drug abus*, drug dependence, drug misuse, drug use*, mania, manic, mental disab*, mental disorder*, mental health, mental ill* disab*, mental ill*, mental* health disorder, mood, neuroses, neurosis, neurotic, obsessive* compulsive, OCD, panic, personality disorder*, phobia, psychiatric, psychiatry, psychology*, psychos*, psychotic, schizophreni*, schizotyp*, somatic, somatoform, stress, substance abus*, substance dependence, substance misuse, substance related disorder*, substance use*, and suicid*. The Search terms ‘antidepressant’, ‘anxiolytic’, ‘antipsychotic’ and ‘mood-stabilizer’ were also exploded and used to search for relevant studies. Citations of retrieved studies were exported into EndNote version ×7, and duplicate studies deleted. Reference lists of included articles were subsequently checked for additional articles of interest, and keywords searched on journal articles to obtain relevant studies.

### Inclusion/exclusion criteria

Inclusion/exclusion criteria for identification of all articles on driving with a mental health condition are presented in Table 1. The primary interest of this review focussed on people diagnosed with schizophrenia, stress/anxiety disorder, depression, personality disorder and obsessive compulsive disorder. Studies on sleep disorders, drugs and alcohol misuse and dependence, attention deficit and hyperactivity syndrome and mood/anger/aggression where a specific mental health condition was not identified, were excluded.

**Table 1:** Inclusion/Exclusion Criteria for Identification of Articles

Inclusion Criteria
• All published literature using any research design, as well as commentaries and literature reviews or discussion pieces. • Persons holding a driver’s licence and diagnosed with a mental health condition/s, including: schizophrenia, stress/anxiety disorder, depression and obsessive compulsive disorder. • Studies investigating medication/drugs tested on drivers with mental health conditions if the studies investigated the effects of the medication/drugs on driving ability. • Publications in English and available in full text.
Exclusion Criteria
• Conference proceedings. • Attention Deficit Hyperactive Disorder (ADHD). • Drivers diagnosed with sleep disorders/ insomnia, drugs or alcohol abuse, mood disorders such as anger or aggression, where a mental health condition was not specifically diagnosed. • Drivers experiencing stress or generalised anxiety with no diagnosis, or driving phobia or fear of driving. • Studies investigating medication/drugs for mental health conditions, tested on healthy adults to determine effect on fitness-to-drive.

Two authors (CU and MS) independently screened titles and abstracts of all identified studies, and excluded ineligible studies against the inclusion/exclusion criteria. Disagreements among authors were resolved by discussion and achieving consensus. Full copies of studies were obtained to determine eligibility of articles that were hard to identify solely based on titles and abstracts.

### Quality assessment/evidence based ratings

The classification criteria developed by the American Academy of Neurology (AAN) [34] and by the Centre for Evidence-Based Medicine (CEBM) [35] were used to assign the class or level to rate the strength of evidence, and to assess the quality of the included studies. According to the CEBM, there are five levels of evidence, ranging from I to V (indicating highest to lowest). The AAN presents a system of rating an article by class. The highest evidence is represented by class I and the lowest is represented by class IV (presented in Table 2). Following these criteria, two authors (CU and MS) independently assessed the quality of articles. Again, disagreements were resolved by discussion and achieving consensus.

**Table 2:** AAN Criteria for Rating a Study by Class - Diagnostic Articles [34] - where the ‘diagnosis’ is fitness-to-drive

	Class I	Class II	Class III	Class IV
Rating article by class	Evidence provided by a prospective study in a broad spectrum of persons with the suspected condition, using a criterion standard for the case definition. Test should be applied in a blinded evaluation. All people undergoing the test have the presence or absence of the condition.	Evidence provided by a prospective study of a narrow spectrum of persons (*N* < 100) with the suspected condition, or a retrospective study of a broad spectrum of persons with an established condition by criterion standard, compared with a broad spectrum of controls.	Evidence provided by a retrospective study where either persons with the established condition or controls are of a narrow spectrum (*N* < 100). The reference standard, if not objective, is applied by someone other than the person performing the test.	Any design where the test is not applied in an independent evaluation OR evidence provided by the expert opinion alone or in descriptive case series (without controls).

### Data extraction

Data were synthesised qualitatively, by using a narrative analysis. This method of analysis involved coding information from within individual studies and grouping them into like categories, based on the data provided and the findings of the analysis. Where there was over-lap in information and a study could potentially have been included under more than one of the categories, the study was allocated to the category for which the most data had been provided. Two authors (CU and MS) independently completed data extraction. Publication details, aims of the study, participant details, study design and findings related to the assessment of fitness-to-drive were extracted, summarised and presented in a table format.

## Results

**Fig. 1:**
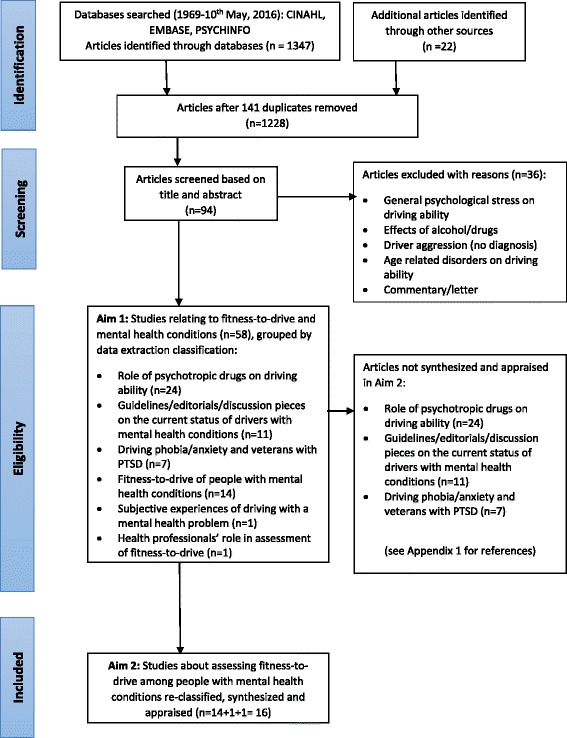
Number of studies identified and screened for inclusion or exclusion

A total of 94 studies were identified after the removal of duplicates (see Fig. 1), and initial screening process. For Aim 1 identifying peer-reviewed, published literature on the topic of mental health and driving, 58 studies were identified and categorised into six subgroups:

1) role of psychotropic drugs on driving ability (*n* = 24), 2) guidelines/editorials/discussion pieces on the current status of drivers with mental health conditions (*n* = 11), 3) driving phobia/anxiety and veterans with PTSD (*n* = 7), 4) fitness-to-drive of people with mental health conditions (*n* = 14), 5) subjective experiences of driving (and fitness-to-drive assessment) with a mental health condition (*n* = 1), and 6) health professionals’ role in assessment of fitness-to-drive (*n* = 1). Further analyses of data from subgroups 1, 2, and 3 were not completed beyond categorising the scope and volume of the literature in this area. The citations for these studies can be viewed in Additional file 1.

For Aim 2 the authors’ critically appraised studies that included data related to assessing fitness-to-drive. Research that primarily focussed on the role of psychotropic drugs, driving phobia/anxiety and veterans with PTSD, and guidelines/editorials/discussion pieces were excluded. While literature focussing on the role of psychotropic drugs is very valuable to understanding fitness to drive, this is a large literature that requires a separate review and detailed background on the medications and their actions and effects in a typical populations as well as populations with mental health problems. The other areas of driving phobia/anxiety and veterans with PTSD, and guidelines/editorials/discussion pieces were excluded as these subgroups did not meet the aim of containing sufficient data related to assessing fitness-to-drive among people with mental health conditions. Fifteen studies were retained for review in Aim 2. As presented in Table 3, three of the 16 included studies were classified as Class III, 12 were classified as Class IV, and one was unclassified since it was secondary analysis of medical records. Under the CEBM classification criteria, the review yielded seven level 4 studies and eight level 5 studies, with the same paper again as unclassified. Among these studies, the majority (*n* = 5) were conducted in Canada. Other studies were conducted in Germany, Ireland, Switzerland, Spain, Denmark and America.

**Table 3:** Summary of key findings and quality appraisal of the 16 included papers, presented by sub-group

Authors (year); location; funding (Y/N)	Aim related to mental health and assessing fitness-to-drive	Participants	Study design/ method/measures	Key findings	Level of evidence (CEBM/AAN)
Sub-group 1: Driving status and/ or factors impacting on the ability to drive safely among people with mental health conditions.					
Brunnauer (2016) [36] Germany; N	To explore the effect of mental illness on driving status in terms of driving licence possession and driving restrictions	*N* = 1859 recruited from five hospitals (*N* = 1546, psychiatric inpatients; *N* = 313, neurological inpatients as control group)	Quantitative: Questionnaire	• Lower proportions of current drivers were found mainly in those with organic mental disorder and psychotic disorder. • 67% of psychiatric patients reported having a valid licence and 77% of this group reported using their cars regularly. • More than 30% of patients with psychotic and organic mental health conditions do not have driving licence despite suitable age and employment status. • The best predictors for having driving licence were education and being partnered or in a relationship, which both reflect psychosocial status. • Female, older age, pension holders, patients with organic mental disease or schizophrenia are associated with increased rates of driving cessation.	CEBM: level 4 AAN: Class IV
Bulmash (2006) [8] Canada; N	To examine the correlation between major depressive disorder (MDD) and driving performance using driving simulator	*N* = 47 (*N* = 18, outpatients diagnosed with MDD, required to be free of antidepressants); *N* = 29, control group)	Quantitative: Naturalistic group comparison Measures: • Epworth Sleepiness Scale (ESS) • Beck Depression Inventory (DBI) • York driving simulator	• The depressed group experienced higher levels of general, on task sleepiness. • Level of sleepiness was statistically associated with driving variables of speed and speed deviation. • The depressed group showed a significantly slower steering reaction time (RT) and greater number of car crashes when compared to controls, which were characterized by a medium effect size. • Slowed RT or increased crash risk were not significantly associated with severity of depressive symptoms. • No statistically significant differences for road position, speed or speed deviation were found when compared to controls.	CEBM: Level 4 AAN: Class IV
De las Cuevas (2008) [37] Spain; N	To examine fitness-to-drive of people with mental health illness	*N* = 208 with psychiatric illnesses	Quantitative: Naturalistic group comparison • LNDETER 100 battery (cognitive functioning and psychomotor performance) • Clinical Global Impressions- Severity of Illness Scale	• Only 33 out of 208 participants were compliant with the requirements of a driver’s licence, and 84% failed at least one of the tests. • Driving patients showed better results than non-driving patients; however, 79.5% still scored too low to obtain or renew their driver’s licence • Only 2 out of 10 professional drivers passed all the tests. • None of the driving patients informed the authority of their mental health status and none stopped driving, although 10% recognized their inability to drive. • In the 6 months prior to the research, only 3 of the 208 patients had been involved in a crash indicating a poor correlation between neuropsychological tests when predicting driving performance	CEBM: level 4 AAN: Class IV
McNamara (2015) [38]; Ireland; N	To explore the experience of driving of people diagnosed with bipolar disorder	*N* = 18, diagnosed with bipolar disorder; holding driving licence; recruited via selective sampling	Qualitative: Thematic analysis of focus group data	• Occurrences of speeding, making poor decisions, losing feeling of control, decreased concentration and judgement, and impulsivity were identified by some participants when driving in manic or hypomanic states. They agreed “no driving” when unwell. • Some participants reported bipolar was unlikely to impede their driving capabilities, compared to the general population. • Every individual reported a unique experience on the effect of bipolar disorder on driving. • Participants reported having an open communication with health professional is useful regarding fitness-to-drive. • The fitness-to-drive guidelines were perceived as discriminatory by drivers with bipolar.	CEBM: level 5 AAN: Class IV
Niveau (2001) [39]; Switzerland; N	To examine if psychiatric patients that were reported to authorities have a higher risk than those were not reported to the police	*N* = 65 with psychiatric disorders (subjects: *N* = 31, reported to authorities / Control: *N* = 34) via purposive sampling	Quantitative: Case control design Measures: • Reason for licence being revoked • Violations • Crashes • Severity of psychiatric disease	• More males and those with lower level of schooling were reported to authorities, than the participants in the control group. • A statistically higher rate of acute disorders was found among the reported cases than the controls. The reported subjects were found to show severe psychiatric antecedents; however, nearly half of them had a clean driving record. • Less than 10% of clean driving records were found in the control group. • Doctors basing their opinion about fitness-to-drive on their own professional criteria would underestimate other factors which could lead to bias in evaluating driving performance.	CEBM: Level 4 AAN: Class III
Segmiller (2015) [40]; Germany; N	To examine psychomotor driving skills of unmedicated first- and recurrent- episode people with schizophrenia, and skill decline with disease progression	*N* = 46 (*N* = 13 first episode and *N* = 13 recurrent episode schizophrenia, and *N* = 20 healthy controls)	Quantitative: Naturalistic group comparison Measures: • Positive and Negative Syndrome Scale • Computerised Weiner Test System (WTS) (visual perception, reactivity and stress tolerance, concentration and vigilance)	• 32% of untreated participants showed severe impairment in ability to complete the tests, which was interpreted as severe impairment in the ability to drive. • A greater percentage of first episode patients (38%), compared to recurrent episode patients (25%), showed pronounced impairments on the WTS. • The problems in psychomotor function seen in both groups could not be attributed to adverse effects of pharmacological treatments. • Both groups of patients with schizophrenia had lower results on the WTS than healthy controls. • Analyses did not reveal a chronological decline in psychomotor function over time (in the early stages of the disease).	CEBM: level 4 AAN: Class III
Zingg (2009) [41]; Switzerland; N	To investigate how performance of driving offenders with a psychiatric diagnosis relevant for driving, driving offenders without a psychiatric diagnosis, and control subjects differed on neuropsychological tests and self-performance on driving	*N* = 219 patients (*N* = 63 with diagnosis relevant to driving; *N* = 111 with diagnosis not believed to affect driving; *N* = 45 controls	Quantitative: Naturalistic group comparison Measures: • Performance test system or Wechsler Intelligence Test • Number connection test • Modified card sorting test (computerized) • Battery for attentional performance	• Participants who were offenders (with and without psychiatric diagnosis) scored significantly lower than the control group on tests of information processing, but only those with a psychiatric disorder also scored significantly lower on problem solving and cognitive flexibility, alertness, and visual scanning/reaction time. • There were no significant differences between the groups on their self-assessment.	CEBM: level 4 AAN: Class IV
Sub-group 2: Capability and perception of health professionals assessing fitness-to-drive of people with mental health conditions.					
Langan (2009) [42]; Ireland; N	To investigate the level of documentation relating to fitness-to-drive in psychiatrists’ clinical notes	*N* = 44 outpatients record, *N* = 48 discharge records; diagnosed with psychiatric illnesses *N* = 11 psychiatrists recruited from acute psychiatric unit	Quantitative: • Secondary data analysis of medical records • Questionnaire	• Minimal documentation on possible effect of illness on driving performance was found in outpatient records and discharged records, 0% and 2% respectively. • More than half of both outpatient (57%) and discharged records (54%) contained documented advice on the importance of compliance with prescribed medication. • 34% of outpatient records and 44% of discharged records contained documentation on the side effects of prescribed medication on driving ability. • No advice regarding medication usage and operation of machinery or driving was found in both sets of records. • Over 50% of psychiatrists indicated they advised patients on the adverse effect of illness or medication on driving performance but few documented this.	CEBM: not applicable AAN: Not applicable
Menard (2006) [43]; Canada; Y	To examine Canadian psychiatrists’ attitudes, practices and knowledge on fitness-to-drive of people with mental health illness	*N* = 248 psychiatrists recruited via random sampling	Quantitative: National cross sectional survey	• 64.1% of respondents strongly agreed or agreed on the importance of addressing fitness-to-drive. • Only 18% of respondents recognised if their patients were active drivers. • One-fourth of respondents strongly agreed or agreed they were capable in assessing fitness-to-drive. • One-half of respondents believed that people with mental illness have a higher risk of a car collision. • Psychiatrists were more likely to advise on the adverse effect of medication on driving, rather than the potential impact of the mental illness itself on driving.	CEBM: level 5 AAN: Class IV
Menard (2012) [44]; Canada; N	To understand the role of occupational therapists in determining fitness-to-drive of people with mental health illness, with a focus on screening, assessment, intervention, perceived barriers and facilitators to undertaking this role	*N* = 20 occupational therapists working in adult mental health recruited via purposive sampling	Qualitative: Phenomenological analysis of focus group data	• An important role for occupational therapists was perceived, with regards to assessing driving ability and raising issues of driving with healthcare team and client. • Limited evidence was found to exist to support practice. • Some participants suggested discussing driving history and habit with their clients; understanding client’s perception of driving abilities and factors impacting on their driving capabilities; and identifying goals related to driving can ensure driving safety.	CEBM: Level 5 AAN: Class IV
Rouleau (2010) [45]; Canada; N	To explore factors that health professionals and clients considered in relation to their fitness-to-drive and recommendations psychiatrists provided after the assessment	*N* = 72 (*N* = 48, drivers, *N* = 24, non-drivers), with a mental health diagnosis recruited via purposive sampling	Quantitative: Survey	• 27.7% of participants indicated illness and medications affect driving performance due to lower concentration (43.9%) and fatigue (29.4%). • 28% of participants indicated illness was related to nervousness, 14.2% reported feeling unstable, 9.5% reported being more careful on the road, and 14.2% reported being more aggressive. • Participants indicated that side effects of medication can cause dizziness (10%), nervousness (5%) and other general negative effect (15%). • Psychiatrists focused on the impact of mood on driving and overall clinical assessment to determine a person’s fitness-to-drive, while occupational therapists focused more on physical status, impulsivity levels, and driving history. Both professionals also considered medication side effects, psychiatric symptoms, cognition and judgement when assessing fitness-to-drive • Occupational therapists administered pen and paper test when assessing driving ability with people with mental health issues; however, the finding was not significant in predicting road performance due to small numbers.	CEBM: Level 5 AAN: Class IV
Vrkljan (2015) [46]; Canada; Y	To identify assessment tools and procedures used by assessors to determine fitness-to-drive, outcomes and recommendations using four case scenarios inclusive of one scenario of a 33 year old woman with schizophrenia (case#4)	*N* = 46 assessors in Canada	Quantitative: National Questionnaire	• 33 assessors (71.7%) would assess driving ability for a client with schizophrenia. • Cognition was identified as a primary issue by 71% of the respondents for a client with schizophrenia. • 17.6% of assessors stated that medication needed to be taken into consideration. • 87.1% of assessors indicated using at least one observational test, followed by a physical (80.6%), perceptual motor (77.4%), or cognitive test (67.7%). • 81.3% of respondents would conduct an on-road assessment. • More than half of the respondents would proceed with licence reinstatement with ongoing monitoring if the client did well on both the off and on-road assessment. • If the client passed the on-road assessment but failed the off road assessment, 19 respondents would recommend return to driving, with 8 would consider reassessment in 6 months.	CEBM: Level 5 AAN: Class IV
Sub-group 3: Crash rates.					
Crancer (1969) [47]; USA; N	To examine the possible link between particular mental health illnesses (psychotic disorders, personality disorders, and psychoneurotic disorders) and driving performance	*N* = 271 psychiatric patients with valid driver licence (*N* = 97 with psychotic disorders, *N* = 79 with personality disorders; *N* = 95 with psychoneurotic disorders) compared to a *N* = 687,228 control group	Quantitative: Epidemiological analysis of secondary data Measures: • Crashes • Violations	• Statistically higher crash rates were identified in both the personality (114% higher) and psychoneurotic groups (49% higher), than the control group. • Crash rates in the schizophrenic group were similar to the control group. • Each of the mental illness groups showed a statistically higher violation rates than the control group, particularly in reckless driving, negligent driving and driving with defective equipment.	CEBM: level 4 AAN: Class III
Eelkema (1970) [48]; USA; N	To examine the correlation between mental illness and crashes	*N* = 238, driving records from a hospital (1960); with matched comparison group (*n* = 290)	Quantitative: Epidemiological analysis of secondary data Measures: • Crashes • Violations pre and post hospitalisation	• Patients discharged from the hospital showed higher crash and violation rates per year. • Greater crash ratio (>1) was found in people with psychosis and psychoneurosis; however, these groups had better records compared to the matched comparison group post discharge (crash ratio < 1). • The highest crash rate was identified in personality disorders, with minimal improvement post discharge. With treatment, crash rates decreased in all categories except for personality disorders. • Males with psychosis and psychoneurosis, and personality disorders showed a rising trend in violations.	CEBM: level 5 AAN: Class IV
Kastrup (1977) [49]; Denmark; N Kastrup (1978) [50]; Denmark; N	Both studies used the same data set: Part 1 (1977): to examine characteristics of psychiatric patients that were involved in crashes Part 2 (1978): To investigate crashes psychiatric patients were involved in and its casualty severity	*N* = 2076 psychiatric patients involved in crashes, compared to *N* = 40,232 involved in all crashes	Quantitative: Epidemiological analysis of secondary data Measures for both: Crashes	Part 1 (1977): • As a group, people with psychiatric disorders accounted for 11% of the crashes in Denmark, and this included pedestrian and vehicle crashes. • Approximately 6% of people who were killed or injured in 1973 in car or pedestrian crash had a psychiatric diagnosis. Part 2 (1978): • Drivers with a psychiatric disorder, who were involved in crashes, including pedestrian and vehicles: - were over-represented by women; and - were more frequently in the age group of 25–54 years. • When compared to the normal population, greater proportions drove stolen vehicles without a valid licence and without seat belt at the time of crash, and were more frequently intoxicated.	CEBM: level 5 AAN: Class IV

Author: Only the first author is included in the table for ease of reading

Location: Country for data collection

Funding: Direct funding received for the study, any personal funding received by the authors was not considered

*AAN* American Academy of Neurology [34]

*CEBM* Centre for Evidence-based Medicine, developed by Oxford CEBM Levels of Evidence working group [35]

*WTS* Weiner Test System

The 16 studies were re-classified based on the data each study provided about assessing fitness-to-drive as follows: 1) factors impacting on the ability to drive safely among people with mental health conditions (*n* = 7) [8, 36–41] capability and perception of health professionals assessing fitness-to-drive of people with mental health conditions (*n* = 5) [42–46] and 3) crash rates (*n* = 4) [47–50].

### Factors impacting on the ability to drive safely among people with mental health conditions.

For those with major depressive disorder, Bulmash et al. [8] found that people with depression, even when not on anti-depression medication, experienced higher levels of sleepiness when driving. This was found to impact their capacity to maintain appropriate speed when driving in a simulator. In addition, it was found that people with depression showed a significantly slower steering reaction time, and a greater number of crashes, as compared to controls. The level of slowed reaction time was not related to the level of severity of symptoms. When not sleepy, road position, speed and speed deviation were no different to those of controls. The association with depression was therefore apparent, even when participants were not on medication, and seemed to manifest itself when sleepiness was experienced in this study.

For those with bi-polar disorder who had committed driving offences, Zingg, Puelschen, and Soyka [41] found that drivers had impairments in problem solving and cognitive flexibility, alertness, and visual scanning/reaction time. It was noted that these signs and symptoms may have been caused by the clinical condition itself, or by medication taken for the condition in this study. Additionally, although information processing ability was found to be impaired in those with bi-polar disorder, this was no more so than in those who had committed driving offences, and who did not have a psychiatric condition. From this study it was suggested that a bi-polar disorder did not appear to have impaired information processing ability specifically. Decrements in psychomotor skill were also reported among people with schizophrenia by Segmiller et al. [40]. However, they demonstrated that reduced skill was not related to medication side effects.

For those in manic and hypomanic states, McNamara and Buckley [38] found that some participants reported speeding, making poor decisions, losing the feeling of control, decreased concentration and judgement, and impulsivity. As some participants reported that they felt their medical condition did not impair their driving ability, as compared with the general population, lack of insight among participants was also apparent in this study. Although participants reported that having an open communication with health professionals about their driving was helpful, they perceived fitness-to-drive guidelines as discriminatory. The underlying signs of grandeur and lack of insight characteristic of hypomanic and manic states among these participants would fit with this lack of capacity to recognise the gravity of risk to themselves and others, and the potential to feel the guidelines were not respectful of their standing.

In the most recent study by Brunnauer et al. [36], the authors explored the impact of mental illness on driving licence possession and driving restrictions. Higher educational status and being partnered or in a relationship was a key predictor for having a driving licence. People with mental health conditions were also found to be less likely to hold a driving licence or to be currently driving, as compared with controls, particularly for those with organic mental and psychotic disorders.

### Capability and perception of health professionals assessing fitness-to-drive of people with mental health conditions

As can be seen in Table 3, Subgroup 2, the majority of studies examining health professionals’ assessment of fitness-to-drive were conducted in Canada, with the remaining few studies being conducted in the Republic of Ireland. The studies examined attention to the mental health condition and its impact on fitness-to-drive, and the nature and occurrence of recording of such factors in the medical notes. Variations in differing health professionals’ foci of interest were also investigated with all studies being exploratory and qualitative in nature.

### Crash rates

Crancer and Quiring [47] conducted the earliest studies examining the link between mental illness and limitations in driving capacity. They identified that those with psychotic disorders, personality disorders and psychoneurotic disorders had higher rates of reckless driving, negligent driving and driving with defective equipment. Other studies undertaking epidemiological secondary data analysis showed that crashes were more prevalent among people with mental health conditions and that the injuries sustained are more serious, with someone with a mental health condition being more likely to be hospitalised than those without [49]. With regards to the diagnostic groups in these studies, in the second stage analysis of the study conducted by Kastrup, Dupont, Bille, and Lund [50], the most common diagnostic group was found to be personality disorder. The drivers with psychiatric diagnosis were found to be characterised by women, aged 25–54 years. They were more frequently alcohol intoxicated, and with a higher blood alcohol level. They were found to be more likely to be driving a stolen vehicle, without a valid licence, and were found not to have used safety belts at the time of the crash. From this study it appears that those with personality disorders are having crashes due to the behavioural manifestations of their condition; that is, they undertake risky behaviours that impact on their ability to drive safely. Elkema, Brosseau, Koshnick, and McGee [48] also reported the highest crash rate of those with mental health conditions was those with personality disorders. In this study, it was noted that the behavioural traits demonstrated by those with personality disorders were very different in substance to those with significant mental illness due to depression and bi-polar disorders, associated with an impairment of executive level function.

## Discussion

The discussion presents an overview of the findings in relation to the two aims, as well as considering limitations of this systematic review and directions for further research. Initially, this systematic review sought to identify and categorise all peer-reviewed, published literature on the topic of mental health and driving. A relatively small literature was identified, with the largest category related to the impact of psychotropic drugs on driving ability. The second largest category of papers related to assessment of fitness-to-drive, and this topic was further explored in relation to ‘impact of specific mental health conditions on the ability to drive safely’, ‘health professionals’ role in assessing fitness-to-drive’, and ‘crash statistics’.

### Impact of specific mental health conditions on the ability to drive safely

Research to date appears to suggest that the behavioural, cognitive and psychomotor impairments that reduce fitness-to-drive may differ between different diagnostic groups. Crancer and Quiring [47] conducted one of the earliest studies on the link between mental health conditions and driving capacity, associating psychotic disorders, personality disorders and psychoneurotic disorders with impaired fitness-to-drive. Due to the age of this study, these findings may need to be reinvestigated as improvements in medication and healthcare for people with these conditions may alter the findings. However, in a recent study Segmillar et al. [40], also found patients with schizophrenia had impaired psychomotor function that could not be attributed to side effects of psychopharmacological treatment, although no chronological decline was found in the early stages of the disease. Similarly, Brunnauer and Laux [51] concluded that even when stabilised with antipsychotic medication, a great proportion of schizophrenic patients are not fit to drive.

For those with major depressive disorder, Bulmash et al. [8] reported higher levels of sleepiness when driving, irrespective of medication use. Similarly, Wingen et al. [9] also concluded that impaired driving performance in this population is probably not due to antidepressant medications. This finding of a high level of sleepiness suggests that behavioural approaches to keep alertness levels higher may need to be considered, such as shortening routes, not eating a heavy meal before driving, and having a caffeine drink half an hour before driving. These interventions could be tested in a simulator experiment to see which elements are most effective for people with major depressive disorders. When comparing the results from Bulmash et al. [8] and Zingg, Puelschen, and Soyka [41], it appears that major depressive disorder is associated with a more widespread impact on the skills needed for driving than those with bi-polar disorder. Zingg, Puelschen, and Soyka [41] found that bi-polar disorder did not impair information processing and driving ability specifically; but rather that poor information processing maybe an underlying weakness of those who commit driving offences. This hypothesis could be researched by introducing information processing testing as part of a standard driving test, and then conducting prospective research to determine the threshold that relates to safe driving.

Another issue raised for drivers with bi-polar disorder relates to self-insight, which resonates with the view of those with bi-polar disorder feeling that the fitness-to-drive guidelines are discriminatory. Reduced self-insight is of real concern for drivers in hypomanic and manic states, who may have ideas of grandeur and reduced self-regulation. Reduced self-insight and self-regulation may be an issue for those with bi-polar disorder requiring further attention in this population specifically, as these capacities do not appear to be impaired by all mental health conditions. The self-report of symptoms affecting capacity to drive identified by Rouleau, Mazer, Menard, and Maryse [45] matched some of those factors measured in a driving simulator, in that people with mental health conditions (predominantly mood disorders) recognised that their condition and medication caused them to have poor concentration, fatigue, dizziness, and sometimes feelings of aggression and nervousness. They reported being more careful when driving or not driving at all when experiencing these symptoms. For those individuals with a mental health condition currently not driving, even though they possess a licence, this suggests a component of self-regulation. The evidence presented by De Las Cuevas and Sanz [37] supported the fact that some drivers with mental health conditions do self-regulate, but only up to a point, since none of the drivers reported their mental health condition to licensing authorities. The issue of non-reporting may be linked with reduced insight about the need to do so or with the associated fear of licence suspension or cancellation.

### Health professionals’ role in assessing fitness-to-drive

Alongside licensing authorities, medical professionals, occupational therapists and psychologists are involved in licensing decisions for drivers with mental health conditions. Menard et al. [43] found that only one quarter of psychiatrists felt skilled in making fitness-to-drive decisions. Half of these psychiatrists felt that people with mental health conditions were more likely to be involved in a crash. Studies have examined the rate of recording of assessment of fitness-to-drive by these professionals, as well as the documentation of advice on managing medication and its impact on driving. In one study, only half of those patients needing advice on the impacts of medication on their ability to drive, had this noted in their medical records. Additionally, there was minimal recording of how the mental health condition itself would impact on the patient’s driving capacity [42]. Over half of these psychiatrists said that they had advised on this issue, but it was not evident in the medical notes. Similar results were found by Menard et al. [43], who noted that psychiatrists were only aware of whether their patient was an active driver in a quarter of cases, and for these patients, psychiatrists were more likely to advise on medication impacts than effects of the medical condition. Rouleau, Mazer, Menard, and Maryse [45] also noted that psychiatrists paid attention to medication side effects on fitness-to-drive, alongside mood.

While psychiatrists may focus on the impact of medication on fitness-to-drive, occupational therapists are able to take clients for on-road driving tests and have been noted to emphasise the driver’s physical status, cognitive skills, impulsivity levels and driving history [44, 46] to assist determine fitness-to-drive. Focussing on impulsivity levels may be regarded as important, since impulsivity can be related to risk taking behaviours and difficulty self-regulating; characteristics present in the profile of those drivers most likely to crash [38, 48, 49]. Menard et al. [44] additionally found that occupational therapists paid attention to the client’s perception of their driving ability, factors impacting on their driving capabilities, and identifying goals related to driving. The emphasis was therefore on the person’s meta-cognitive functions related to self-regulation, risk taking behaviour and on-road driving ability, rather than on the effects of medication or the medical condition itself. Similar results were reported by Vrkljan, Myers, Blanchard, Crizzle, and Marshall [46], with occupational therapists in the study also in favour of the use of an on-road assessment to inform decision making in clients with a mental health conditions. The on-road assessment was used and seen by most participants as the most valid means of making fitness-to-drive recommendations, over-riding clinic-based test results (including simulation), in cases where the clinic-based test had indicated negative results. With clinic-based tests and simulation results lacking predictive validity when used alone [8], on-road assessment is important to consider with drivers with mental health conditions where there is a doubt about their capacity. As noted above, although literature on ADHD was not included in this review, Jerome et al.’s., [26] systematic review of the ADHD and driving literature also suggest that risk taking is an important area for further investigation. In particular, driving anger and aggression have been consistently associated with risky driving, and therefore health professionals can work off-road with clients to develop flexible strategies to better manage these emotions.

### Crash statistics

A number of studies examining the correlation between mental health conditions and risk of crash have shown an association between significant mental illness and primarily higher level cognitive functions, when drivers were tested in a simulator. For example, Bulmash et al. [8] showed that those with major depressive disorders showed slower steering reaction times and a greater number of car crashes when compared to controls using a driving simulator. Driving simulation experiments appear to show that impaired executive functions impact on driving capacity, yet this is not always borne out in crash data held by licensing authorities [39]. One group that does seem to be over-represented in crash rate data, however, is those with personality disorders. Crancer and Quiring [47] confirmed variation in impairment and crash rates, due to diagnostic group. When compared to controls, statistically higher crash rates were identified in both the personality disorder group (114% higher) and in the psychoneurotic group (49% higher), whereas crash rates in the schizophrenic group were similar to the control group. Of note are the findings of Eelkema, Brosseau, Koshnick, and McGee [48] who showed that with treatment, crash rates decreased for all mental health conditions, apart from those with personality disorders. This is an interesting finding to compare against people with ADHD. While people with personality disorders and ADHD demonstrate several of the same traits such as inattentiveness and impulsiveness, in drivers with ADHD, the negative driving outcome are more focussed on driving violations and citations rather than crashes [26]. Approaches to improving safe driving behaviour in those with personality disorders and ADHD require further investigation.

Licensing authority actions concerning renewal, suspension, or cancellation of a person’s driving licence appear to be closely tied with evidence of crashes. Drivers who do not self-report mental health condition/s may come to the attention of licensing authorities after being involved in one or more crashes, and a relationship appears to exist between involvement in a crash and having a driving licence revoked among people with mental health conditions. However, this relationship may not be warranted. For example, in a case control study by Niveau and Kelley-Puskas [39], people reported to the authorities due to severity of psychiatric disease, were actually more likely to have a clean driving record in relation to crashes and violations than those who were not reported. Hence license revocation seemed to be due to the perceived severity of psychiatric disease itself rather than capacity to drive safely. The judgment of fitness-to-drive in this study was based on clinical or medical records, which is far less accurate that on-road assessments. As noted above, further studies investigating how fitness-to-drive recommendations are made for people with mental health conditions are warranted, and would reveal any inherent biases. Of course, licensing authority staff and health professional may always err on the side of caution, as although revoking a person’s driving licence can limit quality of life and subsequent health of the driver, this has to be balanced against allowing an unsafe driver to continue driving. The key is determining whether the driver with a mental health condition is any more unsafe than other drivers, and there is currently insufficient evidence to guide this decision.

### Limitations and directions for future research

This systematic review was limited by the English language restriction imposed, as several studies published in other languages, such as German, could not be accessed. A meta analysis was not possible since studies were generally of low quality and heterogeneous in terms of aims and mental health conditions included. For example, while there is preliminary evidence to suggest that psychomotor skill levels and crash rates are different for populations of drivers with major depressive disorders, schizophrenia, bi-polar disorder and personality disorders, too few studies exist to allow for analyses and recommendations to be made. In contrast, such meta –analyses have been undertaken by pooling study data in the area of drivers with ADHD [26]. Future studies also require larger populations of each diagnostic group so that the impact of key variables on fitness-to-drive outcomes such as years of driving experience, medication use, and severity of the condition, can also be investigated. A systematic review of the 24 papers located that investigate the impact of medication use on drivers with mental health conditions is also required. This new review could also compare findings against comparable studies from the current review that did not specifically investigate the impact of medication on driving. The quality of future studies could also be increased by including age and sex-matched controls where possible, and the inclusion of longitudinal follow-up of people with mental health conditions who both do and don’t drive. It is generally the driver’s duty to report to the licensing authority any permanent or long-term condition which may impact on their fitness-to-drive. With regards to mental health conditions where a person’s insight may be affected, drivers may not be able to detect a problem and may continue to drive when they are not safe to do so. When individuals continue to drive when they are not fit to do so, this creates legal and ethical issues for treating health professionals, as well as the affected individual’s family.

This review has demonstrated that a number of health professionals feel ill-equipped to make fitness-to-drive recommendations for people with mental health conditions. General medical practitioners are expected to conduct assessments to identify individuals who may be unfit to drive, as well as to provide information to the driver licensing authority on an individual’s diagnosis, treatment and extent of impairment. Numerous studies have indicated that few general medical practitioners have any formal training, specific to fitness-to-drive [52–54], although a recent concerted national education programme on medical fitness to drive has proved effective for Irish general practitioners [55]. Furthermore, while specialist driver-assessor occupational therapists do play a role in assessing fitness-to-drive of a wide range of people with heath concerns and age-related health declines [55], the literature provides limited information about their attitudes, practices and knowledge regarding fitness-to-drive among individuals with mental health conditions [44]. Finally, it may be the case that recommendations of fixed time limits for driving cessation imposed on people with mental health conditions may be unrealistic, lack an evidence base and are possibly ignored by health professionals. Whether to suspend, cancel, renew, or issue a person’s driving licence has considerable impact on not only a person’s health, but also on the subsequent health and well-being of all road users. Currently, insufficient evidence exists to support fair and equitable decision making for fitness-to-drive among people with mental health conditions, and further research is required to support health professionals in this area.

## Conclusions

This systematic review has identified a small and disjointed literature in the area of mental health conditions and driving. A narrative appraisal of literature in the area of assessment of fitness-to-drive revealed only 16 studies, excluding those papers focusing on the impact of medication on driving, from which conclusions cannot be reliably drawn. Some of the major limitations associated with these studies include small sample sizes with low numbers in specific diagnostic groups, and a lack of methodological control. Despite these shortcomings, the review indicated the main issues to be considered in relation to determining fitness-to-drive for people with mental health conditions are; the fluctuating nature of the condition and associated symptoms, driver level of insight, behaviour, increasing evidence suggesting a decrement of psychomotor and cognitive skills among people not related to medication use, and a lack of research evidence to support health professionals to make fitness-to-drive recommendations. In conclusion, this systematic review has identified some of the factors impacting on fitness-to-drive for individuals with mental health conditions as well as some predictors of crash. Further research is urgently required to longitudinally investigate skills among drivers from different diagnostic groups, determine strategies that can successfully assist drivers with mental health conditions to better self-regulate as their condition fluctuates, and support the capability of health professionals to assess fitness-to-drive of people with mental health conditions.

## Additional file

Additional file 1: Citations details of studies included in Aim 1 of the study. (DOCX 17 kb)
