# Supplementary material for: A systematic review of evidence for fitness-to-drive among people with the mental health conditions of schizophrenia, stress/anxiety disorder, depression, personality disorder and obsessive compulsive disorder
Source: BMC Psychiatry. 2017 Aug 31;17:318. doi: 10.1186/s12888-017-1481-1 (PMC5579945; doi:10.1186/s12888-017-1481-1)
Supplement: Additional file 1: — Citations details of studies included in Aim 1 of the study. (DOCX 17 kb) [file 12888_2017_1481_MOESM1_ESM.docx]

**Additional file 1
Citations of Studies Included in Aim 1:
Role of psychotropic drugs on driving ability (n=24); Guidelines/editorials/discussion pieces on the current status of drivers with mental health conditions (n=11); Driving phobia/anxiety and veterans with PTSD (n=7)**

**Role of psychotropic drugs on driving ability (n=24)**

Barbone, F., McMahon, A.D., Morris, A.D., Reid, I.C., McDevitt, D.G., MacDonald, T.M. (1998). Association of road-traffics accidents with benzodiazepine use. *The Lancet, 352(9137),* 1331-1336

Brunnauer, A., Buschert, V., Fric, M., Distler, G., Sander, K., Segmiller, F., Zwanzger, P., Laux, G.(2015). Driving performance and psychomotor function in depressed patients treated with agomelatine or venlafaxine. *Pharmacopsychiatry, 45(2),* 47-50.

Brunnauer, A., Laux, G., Geiger, E., Soyka, M., Moller, H-J. (2006). Antidepressants and driving ability: Results from a clinical study. *Journal of Clinical Psychiatry, 67(11),* 1776-1781.

Brunnauer, A., & Laux, G. (2012). Driving ability under sertindole. *Pharmacopsychiatry, 67(11),* 1776-1781.

Brunnauer, A., Laux, G., Zwick, S. (2009). Driving simulator performance and psychomotor functions of schizophrenic patients treated with antipsychotics. *European Archives of Psychiatry and Clinical Neuroscience, 259(8),* 483-489.

Brunnauer, A., Laux, G., Geiger, E., Moller, H-J. (2004). The impact of antipsychotics on psychomotor performance with regards to car driving skills. *Journal of Clinical Psychopharmacology, 24(2),* 155-160.

De Gier, J.J., Hart, B., Nelemans, F.A., Bergman, H. (1981). Psychomotor performance and real driving performance of outpatients receiving diazepam. *Psychopharmacology, 73(4),* 340-344.

De las Cuevas, C., Ramallo, Y., Sanz, E.J.(2010). Psychmotor performance and fitness to drive: The influence of psychiatric disease and its pharmacological treatment. *Psychiatry Research, 176 (2-3),* 236-241.

Grabe, H. J., Wolf, T., Gratz, S., Laux, G. (1998). Polypharmacological Antidepressive Treatment on Central Nervous Information Processing of Depressed Patients: Implications for fitness to drive. *Neuropsychobiology, 37(4),* 200-204.

Grabe, H. J., Wolf, T., Gratz, S., Laux, G. (1999). The influence of clozapine and typical neuroleptics on information processing of the central nervous system under clinical conditions in schizophrenic disorders: Implications for fitness to drive. *Neuropsychobiology, 40(4),* 196-201.

Judd, L.L. (1985). The effect of antipsychotic drugs on driving and driving related psychomotor functions. *Accident Analysis and Prevention, 17(4),* 319-322.

Kagerer, S., Winter, C., Moller, H-J., Soyka, M.(2003). Effects of haloperidol and atypical neuroleptics on psychomotor performance and driving ability in schizophrenic patients. *Neuropsychobiology, 47(7),* 212-218.

Meuleners, L.B., Duke, J., Lee, A.H., Palamara, P., Hildebrand, J., Ng, J.Q. (2011). Psychoactive medications and crash involvement requiring hospitalization for older drivers: A population-based study. *The American Geriatrics Society, 59(9),* 1575-1580.

Orriols, L., Wilchesky, M., Lagarde, E., Suissa, S. (2013). Prescription of antidepressants and the risk of road traffic crash in the elderly: A case-crossover study*. British Journal of Clinical Pharmacology, 76(5),* 810-815.

Ramaekers, J.G., Ansseau, M., Muntjewerff, N.D., Sweens, J.P., O’Hanlon, J.F. (1997). Considering the P450 cytochrome system as determining combined effects of antidepressants and benzodiazepines on actual driving performance of depressed outpatients. *International Clinical Psychopharmacology, 12(3),* 159-169.

Segmiller, F.M., Buschert, V., Laux, G., Nedopil, N., Palm, U., Furjanic, K., Zwanzger, P., & Brunnauer, A., (2015). Driving skills in unmedicated first‑ and recurrent‑episode schizophrenic patients. *European Archives of Clinical Psychiatry and Clinical Neurosciences,* DOI 10.1007/s00406-015-0647-4

Segmiller, F.M., Hermisson, I.M., Riedel, M.M., Seemuller, F.M., Volkamer, T.M., Laux, G.M., Moller, H.M., Brunnauer, A.M. (2013). Driving ability according to German Guidelines in stabilized bipolar I and II outpatients receiving Lithium or Lamotrigine. *Journal of Clinical Pharmacology, 53* (4), 459- 462.

Shen, J., Moller, H., Wang, X., & Shapiro, C.M. (2009). Mirtazapine, a sedating antidepressant, and improved driving safety in patients with major depressive disorder Journal of Clinical Psychiatry, 70 (3), 370-7.

Soyka, M., Kagerer, S., Brunnauer, A., Laux, G., Moller, H-J.(2005). Driving ability in schizophrenic patients: Effects of neuroleptics. *International Journal of Psychiatry in Clinical Practice, 9(3),* 168-174.

Soyka, M., Winter, C., Kagerer, S., Brunnauer, M., Laux, G., Moller, H-J. (2005). Effects of haloperidol and risperidone on psychomotor performance relevant to driving ability in schizophrenic patients compared to healthy controls. *Journal of Psychiatric Research, 39(1),* 101-108.

Van Laar, M.W., Volkerts, E.R., Van Willigenburg, A.P.P. (1991). Therapeutic effects and effects on actual driving performance of chronically administered buspirone and diazepam in anxious outpatients. *Journal of Clinical Psychopharmacology, 12(2),* 86-95.

Verster, J.C., Veldhuijzen, D.S., Volkerts, E.R. (2005). Is it safe to drive a car when treated with anxiolytics? Evidence from on-the-road driving studies during normal traffic. *Current Psychiatry Reviews, 1(2),* 215-225.

Wingen, M., Ramaekers, J.G., Schmitt, J.A.J. (2006). Driving impairment in depressed patients receiving long-term antidepressant treatment. *Psychopharmacology, 188(1),* 84-91.

Wylie, K.R., Thompson, D.J., Wildgust, H.J. (1993). Effects of depot neuroleptics on driving performance in chronic schizophrenic patients. *Journal of Neurology, Neurosurgery Psychiatry, 56(8),* 910-913.

**Guidelines/editorials/discussion pieces on the current status of drivers with mental health conditions (n=11)**

Dun, C., Baker, K., Swan, J., Vlachou, V., & Fossey, E. (2015). 'Drive safe' initiatives: An analysis of improvements in mental health practices (2005-2013) to support safe driving. *British Journal of Occupational Therapy, 78(6),* 364-368.

Dun, C., Bull, B.J., Hitch, D., Lhuede, K., Vlachou, V., Swan, J. (2015). Supporting safe driving practices among consumers of mental health services: Guidelines for assessment. *Psychiatric Services, 66(5),* 536-538.

Errasoul, A., & Daly, R. (2013). New guidelines on fitness to drive: What do they mean to psychiatrists and their patients? *Irish Journal of Psychological Medicine, 30(4),* 233-235.

Harris, M. (2000). Psychiatric conditions with relevance to fitness to drive. *Advances in Psychiatric Treatment, 6,* 261-269.

Kumar, S., & Pickering, B. (2001). 'Fitness to drive' in New Zealand: Psychiatric aspects and the clinician's role. *Australasian Psychiatry, 9(1),* 51-54.

Metzner, J.L., Dentino, A.N., Godard, S.L., Hay, D.P., Hay, L., Linnoila, M. (1993). Impairment in driving and psychiatric illness. *Journal of Neuropsychiatry, 5(2),* 211-220.

Petch, E. (1996). Mental disorder and fitness to drive. *The Journal of Forensic Psychiatry, 7(3),* 607-618.

Rubinsztein, J., & Lawton, C. A. (1995). Depression and driving in the elderly. *International Journal of Geriatric Psychiatry, 10(1),* 15-17.

Tillmann, W.A., Hobbs, G.E. (1949). The accident-prone automobile driver. A study of the psychiatric and social background. *The American Journal of Psychiatry, 106(5),* 321-331.

Warner, J. P. (1996). The older driver and mental illness. *International Journal of Geriatric Psychiatry, 11(10),* 859-862.

Wickens, C. M., Smart, R. G., & Mann, R. E. (2014). The impact of depression on driver performance. *International Journal of Mental Health and Addiction, 12(4),* 524-537.

**Driving phobia/anxiety and veterans with PTSD (n=7)**

Alpers, G.W., Wilhelm, F.H., Roth, W.T. (2005). Psychophysiological assessment during exposure in driving phobic patients. *Journal of Abnormal Psychology, 114(1),* 126-139.

Amick, M.M., Kraft, M., McGlinchey, R. (2013). Driving simulator performance of veterans from Iraq and Afghanistan wars. *Journal of Rehabilitation Research and Development, 50(4),* 463-370.

Classen, S., Cormack, N.L., Winter, S.M., Monahan, M., Yarney, A., Lutz, A.L., Platek, K. (2014). Efficacy of an occupational therapy driving intervention for returning combat veterans. *OTJR: occupation, participation and health, 34(4),* 177-182.

Hannold, E.M., Classen, S., Winter, S., Landford, D.N., Levy, C.E. (2013). Exploratory pilot study of driving perceptions among OIF/OEF veterans with mTBI and PTSD. *Journal of Rehabilitation Research & Development, 50(10),* 1315-1330.

Hwang, E.J., Peyton, C.G., Kim, D.K., Nakama-Sato, K.K., Noble, A.E. (2014). Post-deployment driving stress and related occupational limitations among veterans of operation Iraqi Freedom and Operation Enduring Freedom. *American Journal of Occupational Therapy, 68(4),* 386-394.

Kraft, T., Kraft, D. (2004). Creating a virtual reality in hypnosis: A case of driving phobia. *Contemporary Hypnosis, 21(2),* 79-85.

Williamson, A. (2004). A case of driving phobia treated with dissociative imagery. *Contemporary Hypnosis, 21(2),* 86-92.
